# Composite detection rate as an upper gastrointestinal endoscopy quality measure correlating with detection of neoplasia

**DOI:** 10.1007/s00535-021-01790-3

**Published:** 2021-05-02

**Authors:** Marcin Romańczyk, Bartosz Ostrowski, Tomasz Marek, Tomasz Romańczyk, Małgorzata Błaszczyńska, Krzysztof Budzyń, Maciej Bugajski, Mateusz Koziej, Maciej Kajor, Krzysztof Januszewski, Wojciech Zajęcki, Marek Waluga, Marek Hartleb

**Affiliations:** 1 Chair and department of Gastroenterology and Hepatology, School of Medicine in Katowice, Medical University of Silesia, Medyków 14 Street, 40-752 Katowice, Poland; 2 Endoterapia, H-T. Centrum Medyczne, Tychy, Poland; 3 Endoscopy Unit, District Hospitals of Chorzów Trust, Chorzów, Poland; 4 Department of Anatomy, Jagiellonian University Medical College, Cracow, Poland; 5 Department of Pathomophology and Molecular Diagnostic, School of Medicine in Katowice, Medical University of Silesia, Katowice, Poland; 6 Department of Pathomophology, Zakład Diagnostyki Mikroskopowej, Dr Krzysztof Januszewski, Ruda Śląska, Poland; 7 Department of Pathomophology, District Hospitals of Chorzów Trust, Chorzów, Poland

**Keywords:** Esophagogastroduodenoscopy, Gastrointestinal neoplasm, Quality indicator, Upper gastrointestinal tract

## Abstract

**Background:**

Esophagogastroduodenoscopy (EGD) is commonly used diagnostic method with no widely accepted quality measure. We assessed quality indicator—composite detection rate (CDR)—consisting of detection of at least one of the following: cervical inlet patch, gastric polyp and post-ulcer duodenal bulb deformation. The aim of the study was to validate CDR according to detection rate of upper gastrointestinal neoplasms (UGN).

**Methods:**

It was a multicenter, prospective, observational study conducted from January 2019 to October 2019. The endoscopic reports from 2896 symptomatic patients who underwent diagnostic EGD were analyzed. The EGDs were performed in three endoscopy units located in tertiary university hospital, private outpatient clinic and local hospital.

**Results:**

64 UGNs were detected. The mean CDR was 21.9%. The CDR correlated with UGN detection rate (*R* = 0.49, *p* = 0.045). Based on CDR quartiles, operators were divided into group 1 with CDR < 10%, group 2 with CDR 10–17%, group 3 with CDR 17.1–26%, and group 4 with CDR > 26%. Detection rate of UGN was significantly higher in the group 4 in comparison to group 1 (OR 4.4; 95% CI 2.2 − 9.0). In the multivariate regression model**,** patient age, male gender and operator’s CDR > 26% were independent risk factors of UGN detection (OR 1.03; 95% CI 1.01 − 1.05, OR 2; 95% CI 1.2 − 3.5, and OR 5.7 95% CI 1.5 − 22.3, respectively).

**Conclusions:**

The CDR is associated with the detection of upper gastrointestinal neoplasms. This parameter may be a useful quality measure of EGD to be applied in general setting.

## Introduction

Endoscopy is widely available diagnostic examination of great importance in detection of malignant and non-malignant gastrointestinal lesions. The number of annually performed esophagogastroduodenoscopies (EGDs) only in the U.S. is over 6 million [1]. Esophageal and gastric cancers are still significant health problems, as both diseases are within the top ten most common cancers, which led in 2018 to 1.3 million deaths worldwide [2]. Precise inspection of the upper gastrointestinal tract allows detecting premalignant conditions and early cancers that is connected with better survival [3].

Importance of EGD quality has been raised in several endoscopy guidelines [4–6], as 6.4% of esophageal cancers and 9.4% of gastric cancers may be missed in routine EGD [7, 8]. In the above-mentioned guidelines, the quality indicators relate to pre-, intra- and post-procedural activities. Apart from minimum time of the entire examination and inspection time dedicated to Barrett’s esophagus, no definite quality measure, such as adenoma detection rate or cecal intubation rate for colonoscopy, has been established for EGD [9]. At the same time, detection of the neoplasia in the upper gastrointestinal tract could not be treated as quality indicator because of its low prevalence. Therefore, the surrogate parameters should be used to test the endoscopists inspection quality. Recently, endoscopist biopsy rate (EBR) has been proposed as applicable quality measure [10]. This parameter has been validated for gastric cancer and in expert hands, was related to lower risk of missed lesions.

The aim of our study was to validate a novel quality metrics based on operator’s perception, named “composite detection rate” (CDR). CDR is based on the detection at least one of three mostly benign lesions: esophageal inlet patch, gastric polyp and post-ulcer duodenal bulb deformation. These findings were chosen as localized in all three investigated segments during EGD, fairly stable and no too low prevalence, not influenced by definition and requiring careful examination and/or special maneuvers. We assumed that search for those lesions pushes investigator to maximize focus on complete and exact inspection of three segments of upper digestive tract. We also hypothesized that thorough examination resulting in higher CDR is related to the detection of neoplastic lesions of the upper digestive tract.

## Materials and methods

### Study design

It was a prospective, multicenter, observational study conducted from January 2019 to October 2019. The study was performed in three centers: endoscopy unit of tertiary university hospital (center A), private outpatient endoscopy clinic (center B) and endoscopy unit of district hospital (center C). The study was exempt from institutional board review (reviewed by Ethic Committee of Medical University of Silesia decision no KNW/0022/KB/235/18). The study was conducted in accordance with the Declaration of Helsinki.

**Fig. 1:**
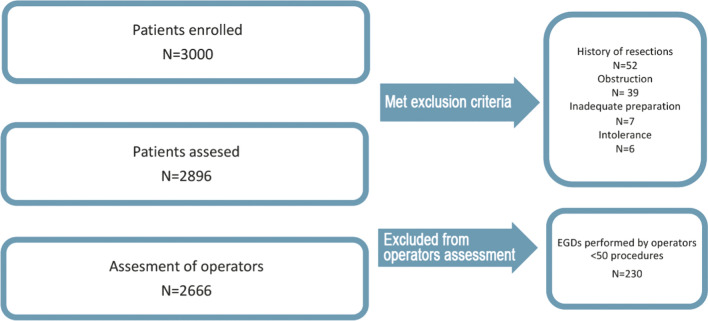
Patient enrollment

One thousand subsequent adults form each site, who were found eligible for elective diagnostic EGD, were enrolled to the study. The EGDs were performed by gastroenterologists, internal medicine or general surgery specialist after completed endoscopic training. Operators were aware of being observed of their quality of performance but were not informed what findings were assessed. Urgent and therapeutic procedures were excluded. Patient enrollment flowchart is summarized in Fig. 1.

The recorded parameters were:

• Patient data: sex, age, indication for endoscopy.
• Procedure information: in/outpatient procedure, sedation, type of endoscope, name of the operator, biopsies taken.
• Endoscopy report findings: esophageal inlet patch, reflux esophagitis, Barrett’s esophagus, esophageal polyp, esophageal tumor, gastritis, gastric polyp, gastric peptic ulcer, gastric tumor, duodenal bulb post-ulcer deformation, duodenal ulcer, duodenal polyp, duodenal tumor.
• Pathology report: Barrett’s esophagus, Barrett’s esophagus related dysplasia and adenocarcinoma, squamous intraepithelial dysplasia and carcinoma, esophageal stromal and neuroendocrine tumor, gastric dysplastic lesions, gastric cancer, gastric lymphoma, gastric neuroendocrine tumor, gastric stromal tumor, duodenal adenoma, duodenal cancer, duodenal lymphoma, duodenal neuroendocrine tumor, and duodenal stromal tumor.

We excluded from assessment patients with incomplete EGD (e.g., upper gastrointestinal obstruction, examination intolerance, inadequate preparation) or history of surgery (esophageal, gastric or duodenal resections). Operators who performed less than 50 procedures were excluded from operator’s assessment. *Helicobacter pylori* infection was diagnosed in case of positive rapid urease test or in histopathological examination.

CDR was calculated as the proportion of patients in whom at least one of the following lesions were detected: esophageal inlet patches, gastric polyp and post-ulcer duodenal bulb deformation. EBR was calculated as the proportion of examinations with at least one biopsy taken.

Main endpoint of this study was upper gastrointestinal neoplasm (UGN) detection rate calculated as proportion of patients whom at least of the following was detected—cancer, non-epithelial tumor and precancerous condition (adenoma, gastric dysplastic lesion, squamous intraepithelial neoplasm, dysplasia in the Barrett’s esophagus).

### Statistical analysis

The quantitative data were reported as mean ± standard deviation (SD) or median/interquartile range, according to distribution status fit. For the qualitative data, frequencies and percentages were calculated. Odds ratios (ORs) and 95% confidence intervals (95% CI) for detection of UGN were calculated in relation to the baseline (group 1) for each of the groups (2, 3, 4) based on CDR. Uni- and multivariate logistic regression analysis were performed to assess the risk factors of detecting UGN (dependent value). Also, Spearman correlation between CDR and EBR of each operator and UGN was performed. Comparisons were performed using t test or Mann–Whitney test for two groups depending on normality. The qualitative variables were compared using the *χ*^2^ test of proportions for categorical variables with Bonferroni’s correction if needed. The data were analyzed using StatSoft Statistica 13.0 PL for Microsoft Windows 10. The results with *p* value < 0.05 were considered as statistically significant. The sample size was estimated based on UGN prevalence that was known to be approximately 2.6% in the tertiary unit (based on the cohort of 1000 retrospectively assessed EGDs in the center A). The power analysis indicated that to detect correlation of *r* = 0.35 utilizing a 2-sided test, 5% significance level test (*α* = 0.05) with 80% power (*β* = 0.2), the required minimum sample size was 61 cases. Therefore, one thousand patients from each center was considered a representative sample.

## Results

### General data

Of 3000 eligible patients, 2896 were analyzed, (104 patients were excluded) – see Fig. 1. Demographic data and endoscopic findings are summarized in Table 1. Mean age of participants was 56.9 years. 66% were outpatients and 34% inpatients, 57.3% were female. Three most common indications for EGD were: gastroesophageal reflux disease symptoms (13.2%), dyspepsia (11.8%) and malabsorption (9%). Almost 60% of procedures were performed on high definition endoscopes.

**Table 1:** Demographic, endoscopic procedure and endoscopic findings data

Demographic data	
Number of patients	2896
Mean age (y ± SD)	56.9 ± 16
Sex (M/F)	1237/1659
Indication for endoscopy [*n*/%]	
Gastroesophageal reflux disease	383/13.2%
Dyspepsia	343/11.8%
Malabsorption	261/9%
Suspicion of malignancy	221/7.6%
Evaluation of portal hypertension	175/6%
Surveillance of gastritis	167/5.8%
Other	1346/46.5%
Procedure information	
Sedation [*n*/%]	968/32.4%
High-definition/non-high-definition endoscopes	1727/1169
In/outpatient	1075/1911
Endoscopic findings [*n*/%]	
Gastric inlet patch	168/5.8%
Reflux esophagitis	480/16.6%
Non-dysplastic Barrett’s esophagus	70/2.4%
Dysplasia in Barrett’s esophagus	14/0.5%
Squamous intraepithelial neoplasia	1/0.03%
Esophageal cancer and neuroendocrine cancer	10/0.4%
Gastritis	1991/68.8%
Gastric polyps	444/15.3%
Gastric dysplastic lesions	8/0.3%
Gastric neuroendocrine tumors	2/0.07%
Gastric cancers	18/0.6%
Gastric peptic ulcer	77/2.7%
Helicobacter pylori infection	289/10%
Dudenal bulb deformation	74/2.6%
Duodenal ulcer	55/1.9%
Dudenal polyps	44/1.5%
Duodenal adenomas	9/0.3%
Duodenal cancer	2/0.07%
Helicobacter pylori infection/tested	381/1486/25.6%
Quality metrics [*n*/%]	
UGN	64/2.2%
Biopsy rate	1870/64.6%
CDR	635/21.9%

*M* Male, *F* female, *HD* high definition, CDR – composite detection rate, *UGN* upper gastrointestinal neoplasm

### Key endoscopic findings

Sixty four UGN were detected of which 14 were dysplasias in the Barrett’s esophagus, 1 Squamous intraepithelial neoplasia, 7 esophageal cancers, 3 esophageal neuroendocrine cancers, 8 gastric dysplastic lesions, 18 gastric cancers, 2 gastric neuroendocrine tumors, 9 duodenal adenomas and 2 duodenal cancers. CDR’s components, i.e. esophageal inlet patch, gastric polyp, and duodenal bulb deformation were detected with rates of 5.8%, 15.3% and 2.6%, respectively. Mean CDR was 21.9%. In 1870 procedures, at least one biopsy was obtained, resulting in biopsy rate of 64.6% (see Table 1).

### Comparison of endoscopists

**Fig. 2:**
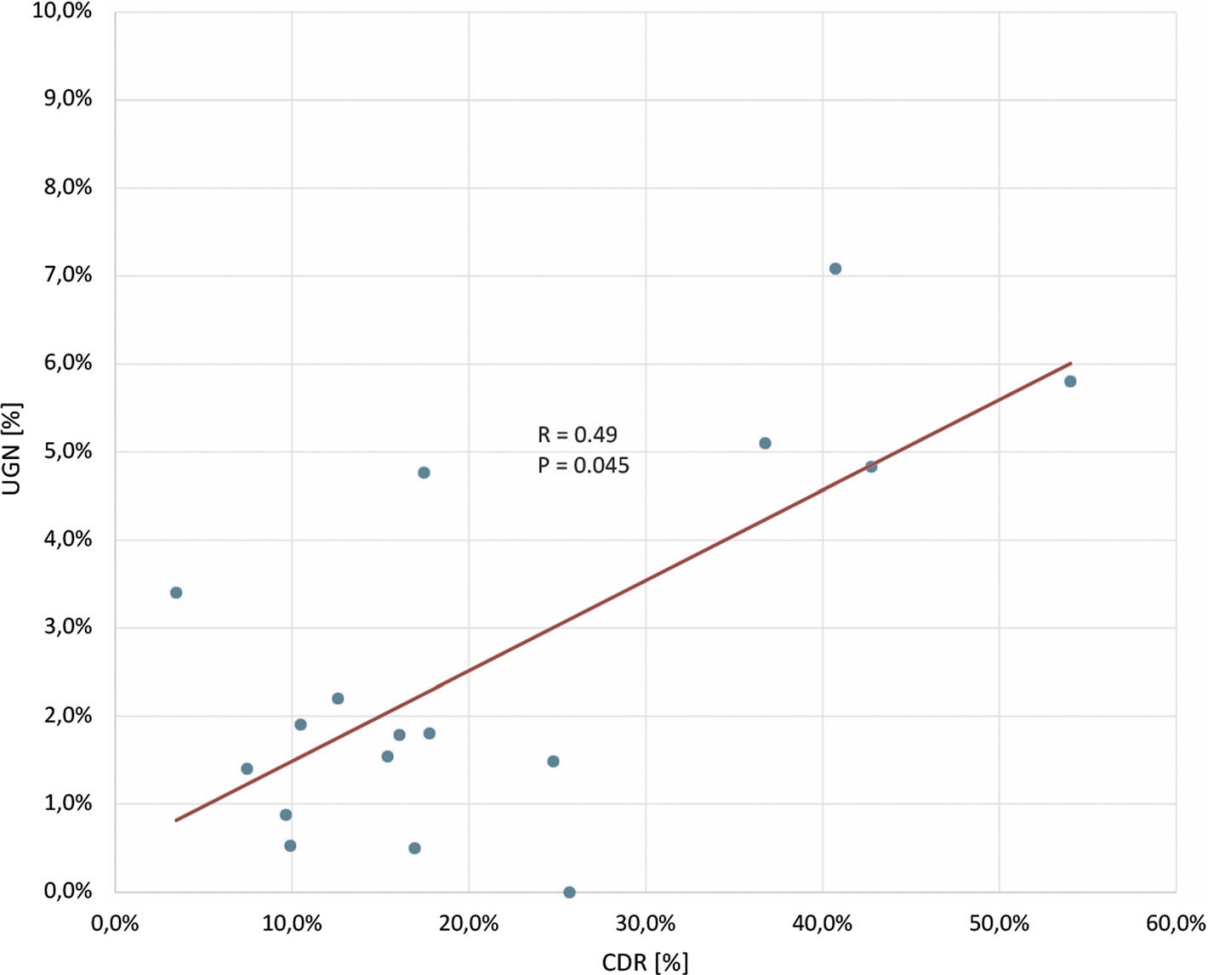
Composite detection rate in relation to upper gastrointestinal neoplastic lesions detection. Each spot reflects an endoscopist. *CDR* composite detection rate, *UGN* upper gastrointestinal neoplasm

Of 28 operators, 17 have performed at least 50 examinations (in total 2666 EGDs). The median CDR for analyzed operators was 17% and individual values ranged from 3.4 to 54%. The operators were divided into four groups based on the CDR quartiles: group 1 CDR < 10% (4 operators), group 2 with CDR 10.0–17.0% (5 operators), group 3 with CDR 17.1–26.0% (4 operators) and group 4 with CDR > 26% (4 operators). UGN detection rate ranged from 0.5 to 7.1% for analyzed operators. Mean UGN detection values were 1.4%, 1.6%, 1.9%, 6% in groups 1–4, respectively. In the logistic regression analysis, the odds ratio for neoplastic lesion detection increased from 1.1 to 4.4 in the groups 2–4 in relation to group 1. The data are summarized in the Table 2. The correlation between CDR and UGN was statistically significant (R = 0.49, *p* = 0.045; Fig. 2).

**Table 2:** Operators performance based on composite detection rate

Operator	Center	CDR [%]	UGN [%]	EBR [%]	CDR group	UGN [%]	OR for UGN detection [95% CI]	*p* value	Biopsy rate
1	A	54.0%	5.8%	81.3%	Group 4 > 26%	6.0%	4.4 [2.2 − 9.0]	< 0.001	80.9%
2	A	42.8%	4.8%	89.7%					
3	A	40.7%	7.1%	61.9%					
4	A	36.7%	5.1%	88.8%					
5	B	25.7%	0.8%	51.3%	Group 3 17.1–26%	1.9%	1.2 [0.5 − 2.8]	0.67	54.7%
6	B	24.8%	1.5%	64.9%					
7	B	17.8%	1.8%	31.4%					
8	C	17.5%	4.8%	98.4%					
9	B	16.9%	0.5%	43.3%	Group 2 10–17%	1.6%	1.1 [0.4 − 2.7]	0.87	64.9%
10	C	16.1%	1.8%	78.6%					
11	A	15.4%	1.5%	53.8%					
12	C	12.6%	2.2%	74.8%					
13	C	10.5%	1.9%	93.3%					
14	C	9.9%	0.5%	83.3%	Group 1 < 10%	1.4%	1	Reference	60.6%
15	B	9.6%	0.9%	36.0%					
16	C	7.4%	1.4%	51.2%					
17	A	3.4%	3.4%	26.7%					

*CDR* composite detection rate, *UGN* upper gastrointestinal neoplasm, *EBR* endoscopist biopsy rate, *OR* odds ratio, *CI* confidence interval

### Risk factors of UGN detection

In the univariate analysis, age, male gender, inpatient procedure, endoscopic center A (in comparison to center B) and EGD performed by endoscopist with CDR > 26% were risk factors of UGN detection. In the multivariate analysis, operators with CDR > 26% were over five times more likely to detect an UGN (OR 5.7; 95% CI 1.5 − 22.3). Age and male gender were also found to be independent factors with—see Table 3.

**Table 3:** Logistic regression analysis of upper gastrointestinal neoplasm detection risk factors

Variables			Univariate			Multivariate		
			OR	95% CI	*p* value	OR	95% CI	*p* value
Age			1.03	1.01 − 1.05	** < 0.001**	**1.03**	**1.01 − 1.05**	**0.002**
Male			2.0	1.2 − 3.4	0.005	**2.0**	**1.2 − 3.5**	**0.011**
HD-endoscope			0.95	0.6 − 1.6	0.858			
Sedation			0.8	0.4 − 1.3	0.326			
Out-patient			0.4	0.2 − 0.7	< 0.001	**0.7**	**0.4 − 1.2**	**0.207**
CDR > 26%			4.6	2.8 − 8	< 0.001	**5.7**	**1.5 − 22.3**	**0.012**
Endoscopic center*		**B**	Reference			**0.8**	**0.3 − 2.0**	**0.666**
		**C**	**1.4**	**0.6 − 3.1**	**0.430**			
		**A**	**4.0**	**2.0 − 8.1**	** < 0.001**			

*HD* high definition, CDR > 26% – examination performed by operator with CDR higher that 26% (group 4), OR – odds ratio, CI – confidence interval

*In the univariate analysis center with lowest mean UGN value (center B) was the reference. In the multivariate analysis the centers were graded according to mean UGN value – center B as 1, center C as 2 and center A as 3

### Comparison of endoscopy centers

Highest CDR was achieved in center A (35.3%, university endoscopy center) and the highest biopsy rate (76.6%) was recorded in center C (endoscopy unit of district hospital) in which CDR was the lowest (10.6%). The differences in CDR and EBR were significant among centers. Overall UGN detection rate was 2.2% and was significantly higher in center A than in B and C (4.2% vs 1% vs 1.5%, respectively; A vs C and A vs B *p* = both < 0.001, B vs C *p* = 0.32). The data are summarized in Table 4.

**Table 4:** Composite detection rate, biopsy rate and upper gastrointestinal neoplasm among centers

	Center A	Center B	Center C
Patients [n]	950	988	958
Biopsy rate [%]	68.5%^a,b^	49.0%^a,c^	76.7%^b,c^
CDR [%]	35.3%^a,b^	20.0%^a,c^	10.6%^b,c^
UGN [*n*/%]	40/4.2%^a,b^	10/1%^a^	14/1.5%^b^

*CDR* composite detection rate, *UGN* upper gastrointestinal neoplasm

^a^Significant difference in comparison center A with center B

^b^Significant difference in comparison center A with center C

^c^Significant difference in comparison center B with center C

*p*-value < 0.017 considered as significant according to Bonferroni’s correction

### Relationship between EBR and CDR

Mean EBR among operators was 64.6% ranging from 26.7 to 98.4%. EBR was highest in the group with highest CDR, however, there was no clear correlation between either EBR and CDR (R = 0.36, *p* = 0.16) or EBR and UGN detection (R = 0.43, *p* = 0.08). Threshold of 43.8% for EBR (validated by Januszewicz et al.) was fulfilled by 13 of 17 operators, however, reaching this threshold level insignificantly improved UGN detection (OR 1.55, 95%CI 0.8 − 3.1, *p* = 0.18) with detection rates 1.5% for EBR < 43.8% vs 2.6% for EBR ≥ 43.8%, *p* = 0.2).

## Discussion

The appropriate quality indicator for EGD has been searched for some time with no tangible success. Difficulties in finding proper EGD quality indicator stem from several reasons. First, unlike in colonoscopy, which is targeted to detect and remove adenomatous polyps, in upper digestive tract the prevalence of precancerous lesions is too low to be treated as quality indicator [11]. Second, during the EGD, three different parts of digestive tract are investigated with different risk of cancer development and different phenotypes of the precancerous lesions. Generally, a quality indicator should be simple, easily analyzed among large numbers of endoscopies performed by different operators in different clinical settings. Several EGD quality metrics have been proposed so far. Procedure time [12–15] and Vater’s ampulla photo-documentation [16] have been recently proposed, however, low-quality data based on single-center investigations demand further validation. The most recently proposed quality metrics is endoscopist biopsy rate (EBR), which was shown to be related to reduced risk of missed gastric cancers [10]. However, there was no correlation between EBR and the detection of gastric dysplasias—conditions predisposing to cancer development [17]. In our study, EBR value did not correlate with UGN detection either, suggesting that some endoscopists might be excessively focused on benign lesions with no clinical relevance. Other factors might also have impact on EBR. Routine biopsies to evaluate chronic gastritis, biopsies indicated by clinical background irrespective of endoscopic findings (for example in diagnosis of coeliac disease) increase EBR without reflection in neoplastic lesions detection. On the other hand, “no touch” optical diagnosis is also highly recommended to avoid fibrosis hindering radical endoscopic treatment. Financial aspect of EBR should also be raised. Pushing endoscopist to obtain a higher number of biopsy samples increases burden of pathomorphology departments with surge of procedure costs without obvious impact on clinical outcome. CDR based solely on endoscopic examination report is free of additional costs. Another phenomenon should also be mentioned. In the endoscopy unit where EBR was highest, the CDR was lowest with lower UGN detection than in the tertiary unit. It may suggest that biopsy without proper inspection does not protect against lesion omission.

Detection of precancerous conditions and early cancers is the one of the main goals of the EGD. Atrophic gastritis and gastric intestinal metaplasia detection rates were mentioned by Park et al. as EGD potential quality indicators, especially in the countries where gastric cancer prevalence is low [13]. However, their clinical significance is uncertain in low-risk gastric cancer populations [18, 19] and have not been validated as quality indicators. We assumed that the role of quality measure might be played by perception level of the endoscopist verified by his performance in finding tiny but more frequently occurring benign lesions. For this purpose, composite detection rate (CDR) was created based on 3 probably underreported endoscopic findings located in 3 different segments of the digestive tract explored in EGD, namely esophageal inlet patches, gastric polyps and duodenal bulb deformations. Esophageal inlet patches occur in general population with frequency of around 10%, but are probably underrecognized [20]. Detection of these lesions were proposed as quality measure for EGD, however, no validation study for use of this parameter was done as yet [21]. It is noteworthy, that detection of tiny gastric polyps requires not only careful mucosal inspection but also thorough mucosal cleansing. Our assumption was that vigilant endoscopic inspection associated in the high detection of these 3 lesions is simultaneously associated with a higher number of detected neoplastic lesions. Therefore, the role of CDR was to measure general attentiveness and carefulness of endoscopist, which in a natural way improves ability to identify all kinds of pathologies within EGD reach. Similar idea of using gastric diverticula or subepithelial lesions has recently been put forward [11]. However, it seems that the prevalence of these lesions is too low to make them proper quality metric.

We prospectively validated CDR in three endoscopy units performing EGDs for in- and out- patients, localized in tertiary hospital, district hospital and private center. Such selection was aiming to cover the whole range of real-world endoscopy practice. The need for validation of quality indicators in different endoscopy units was also stressed by others [22]. Combination of lesion detection in the all parts of upper gastrointestinal tract seems to be appropriate as more than half of neoplastic lesions detected on EGD examinations were located in the esophagus or duodenum (36 out of 64 neoplastic lesions). The major finding of our study was positive correlation between CDR and neoplastic lesion detection rate. Based on our analysis we found that CDR of 26% is related to higher neoplastic lesions detection as well as being independent risk factor of neoplastic lesion detection in the upper gastrointestinal tract. This observation warrants further validation using CDR as quality indicator of EGD to clarify if it is correlated with improved detection of early cancers and lower rate of missed neoplasia. Analysis of missed neoplasm based on interval cancer development will be final confirmation of CDR effectiveness as well as opportunity to set the proper cutoff value.

We have found out that Helicobacter pylori prevalence in the study population was significantly lower than in the previous reports regarding Polish population [23]. It might reflect decreasing Helicobacter pylori prevalence in Poland stressed by others [24]. Also different testing methods, mass eradication effect, test and treat strategy in our study population, unknown status of previous infection treatment and used medications by enrolled patients may influence the lower infection rate.

Our study has several limitations. First, we did not compare CDR with other quality metrics such as examination time or Vater’s ampulla photo-documentation [16]. Relation of CDR and mentioned metrics is an interesting point for the future research. Second, we were not able to validate CDR in the detection of only early lesions due to their low prevalence. Third, the performance of CDR may be different in the different populations such as countries with low prevalence of *Helicobacter pylori* infection (lower incidence of gastritis and duodenitis), low PPI intake rate or screening population. Finally, referral bias was not taken into account in our study. UGN detection rate was the highest in the tertiary center. UGN detection might be higher due to possible patients preselection which could result in higher number of patients with precancerous conditions of high neoplastic lesions development risk (e.g., long Barrett’s esophagus, extensive atrophic gastritis).

In conclusion, CDR is promising quality indicator of EGD showing close relationship with detection of neoplastic lesions.
