# Correction to: Composite detection rate as an upper gastrointestinal endoscopy quality measure correlating with detection of neoplasia

**DOI:** 10.1007/s00535-022-01899-z

**Published:** 2022-06-27

**Authors:** Marcin Romańczyk, Bartosz Ostrowski, Tomasz Marek, Tomasz Romańczyk, Małgorzata Błaszczyńska, Krzysztof Budzyń, Maciej Bugajski, Mateusz Koziej, Maciej Kajor, Krzysztof Januszewski, Wojciech Zajęcki, Marek Waluga, Marek Hartleb

**Affiliations:** 1 Chaiir and Department of Gastroenterology and Hepatology, School of Medicine in Katowice, Medical University of Silesia, Medyków 14 Street, 40-752 Katowice, Poland; 2 H-T. Centrum Medyczne, Tychy, Poland; 3 Endoscopy Unit, District Hospitals of Chorzów Trust, Chorzów, Poland; 4 Department of Anatomy, Jagiellonian University Medical College, Cracow, Poland; 5 Department of Pathomophology and Molecular Diagnostic, School of Medicine in Katowice, Medical University of Silesia, Katowice, Poland; 6 Department of Pathomophology, Zakład Diagnostyki Mikroskopowej, Dr Krzysztof Januszewski, Ruda Śląska, Poland; 7 Department of Pathomophology, District Hospitals of Chorzów Trust, Chorzów, Poland

## Correction to: Journal of Gastroenterology (2021) 56:651–658 10.1007/s00535-021-01790-3

In the original publication of the article, Table 3 has been published with bold font wrongly which should be limited to the statistically significant *p *value (< 0.05). The correct Table 3 is given in this correction.

**Table 3:** Logistic regression analysis of upper gastrointestinal neoplasm detection risk factors

Variables		Univariate			Multivariate		
		OR	95% CI	*p *value	OR	95% CI	*p *value
Age		1.03	1.01–1.05	** < 0.001**	1.03	1.01–1.05	**0.002**
Male		2.0	1.2–3.4	**0.005**	2.0	1.2–3.5	**0.011**
HD-endoscope		0.95	0.6–1.6	0.858			
Sedation		0.8	0.4–1.3	0.326			
Out-patient		0.4	0.2–0.7	** < 0.001**	0.7	0.4–1.2	0.207
CDR > 26%		4.6	2.8–8	** < 0.001**	5.7	1.5–22.3	**0.012**
Endoscopic center*	B	Reference			0.8	0.3–2.0	0.666
	C	1.4	0.6–3.1	0.430			
	A	4.0	2.0–8.1	** < 0.001**			

*HD* high definition, *CDR > 26%* examination performed by operator with CDR higher that 26% (group 4), *OR* odds ratio, *CI* confidence interval

*In the univariate analysis center with lowest mean UGN value (center B) was the reference. In the multivariate analysis the centers were graded according to mean UGN value – center B as 1, center C as 2 and center A as 3

